# Non-invasive contrast enhanced ultrasound molecular imaging of inflammation in autoimmune myocarditis for prediction of left ventricular fibrosis and remodeling

**DOI:** 10.1371/journal.pone.0224377

**Published:** 2019-10-28

**Authors:** David C. Steinl, Lifen Xu, Amanda Ochoa-Espinosa, Mukesh Punjabi, Beat A. Kaufmann

**Affiliations:** 1 Department of Biomedicine, University of Basel, Basel, Switzerland; 2 Division of Cardiology, University Hospital and University of Basel, Basel, Switzerland; Scuola Superiore Sant'Anna, ITALY

## Abstract

**Background:**

Myocarditis can lead to myocyte loss and myocardial fibrosis resulting in dilated cardiomyopathy (DCMP). Currently employed methods for assessing the risk for development of DCMP are inaccurate or rely on invasive myocardial biopsies. We hypothesized that molecular imaging of tissue inflammation with contrast enhanced ultrasound during peak inflammation in myocarditis could predict development of fibrosis and impaired left ventricular function.

**Methods and results:**

Experimental autoimmune myocarditis (EAM) was induced in Balbc mice by injection of the α-myosin heavy chain peptide. Contrast enhanced ultrasound (CEU) using microbubbles targeted to leukocytes (MB_Lc_), to CD4+ lymphocytes (MB_CD4_), and to the endothelial cell adhesion molecule P-selectin (MB_PSel_) was performed during the expected EAM peak inflammatory activity 21 days after induction. High resolution ultrasound, invasive hemodynamic measurements and fibrosis quantification were done 63 days after EAM assessment. All tested microbubbles correlated to fibrosis (MB_Lc_ spearman r 0.28, p 0.047, MB_CD4_ r 0.44, p 0.01, MB_PSel_ r 0.73, p 0.02), however, correlations were weak overall and the spread of data was considerable. Also, targeted CEU data on day 21 did not correlate to hemodynamic and functional data on day 63.

**Conclusions:**

Ultrasound molecular imaging using targeted microbubbles during the peak inflammatory activity of myocarditis correlates weakly with later development of fibrosis but not with hemodynamic or left ventricular functional parameters.

## Introduction

In myocarditis triggered by infection, systemic diseases, drugs or toxins acute inflammation may develop into a chronic autoinflammatory process [1]. Ongoing low-grade inflammation in turn can lead to tissue fibrosis, myocardial remodeling and ultimately to dilated cardiomyopathy (DCMP). Given the diverse etiology of myocarditis and difficulty in diagnosis, the frequency of progression to DCMP is not precisely known. However, in a prospective study examining a cohort of patients with viral myocarditis, around 20% experienced sudden cardiac death or heart transplant during follow up, suggesting development of DCMP in at least a fifth of patients in this particular study [2]. The development of DCMP carries a poor prognosis and can lead to death or the need for heart transplantation [3]. Also, in young adults suffering sudden death, histologic evidence of myocarditis is identified in about 10% [2] of the cases.

The pathogenesis of myocarditis involves acute injury of the myocytes that initiates immune processes with a CD4 T cell response as the main driving force. Multiple factors such as gender, Human Leukocyte Antigen (HLA) haplotype, exposure of encrypted self antigens such as cardiac myosin and molecular mimicry with cross-reactivity of myosin with microbial epitopes contribute to a sustained autoimmune response with T cell and macrophage infiltration of the myocardium [1,4,5].

For the prediction of ultimate development of DCMP, New York Heart Association functional class upon presentation and immunohistological evidence of inflammation have been shown to be related to poor outcome [2]. However, immunohistological analysis depends on endomyocardial biopsies which are invasive and prone to sampling error. Thus, a method for non-invasive assessment of inflammatory activity and the components thereof in the myocardial tissue could potentially be of value in the prediction of DCMP development.

The Experimental Autoimmune Myocarditis (EAM) murine model has been developed to recapitulate and study the pathophysiologic processes involved in acute and chronic human myocarditis [6]. In susceptible mouse strains such as Balb/c mice, autoimmune myocarditis is induced by injecting pertussis toxin and α-myosin heavy chain peptide and thus simultaneously eliciting a cellular immune response and a self-antigen challenge. This protocol results in myocarditis with inflammatory activity peaking around 21 days and development of DCMP around 60 days after induction. In this EAM model, we have previously shown that ultrasound molecular imaging can be used to detect both the peak endothelial inflammatory activation and leukocyte infiltration that take place in autoinflammatory myocarditis. Of note, using microbubbles targeted to the glycoprotein CD4, detection of the recruitment of CD4+ T cells that are crucial in driving the autoinflammatory process that ultimately leads to DCMP was possible [7].

The aim of our study was therefore to assess whether, in addition to diagnosis of the acute disease, ultrasound molecular imaging of the peak autoimmune inflammation in the EAM model can be used to predict future left ventricular structural changes or functional deterioration that are observed in DCMP.

## Material and methods

All data have been made publicly available at the Zenodo repository and can be accessed at 10.5281/zenodo.3356728.

### Study design and animal model

All experiments were performed in accordance with Swiss Federal Legislation and were approved by the Animal Care Committee of Basel. A total of 54 female BALB/c mice (8 weeks of age) were used for this study. Female animals were used as they are less susceptible to develop myocarditis than males and we have shown a wide range of myocarditis severity on histology using female animals previously [7]. EAM was induced by intraperitoneal injection of pertussis toxin (400ng, List Biological Laboratories) followed by subcutaneous injection of αMyHC peptide (120μg, alpha-myosin heavy chain, Ac-RSLKLMATLFSTYASADR-OH, GeneCust) emulsified with complete freund’s adjuvant (CFA, Sigma, 1mg/ml) one hour later on day 0. On day 7, an injection of αMyHC only was repeated. On day 21 after induction, the mice were anesthetised for acquisition of high-resolution transthoracic echocardiography including speckle tracking strain imaging, and contrast enhanced ultrasound (CEU) molecular imaging for leukocytes, P-Selectin and CD4+ lymphocytes. Following the imaging on day 21, the animals were recovered. 60–65 days post-induction the animals were again anesthetised for high resolution transthoracic echocardiography and speckle tracking strain imaging, and invasive left ventricular pressure measurements were performed. Afterwards, the animals were sacrificed and the hearts were harvested for histology.

### Microbubble preparation

Perfluorocarbon-filled, microbubbles with a lipid shell were prepared by sonicating an aqueous suspension of distearoyl phosphatidylcholine (2mg/ml; Avanti Polar Lipids, Alabaster AL, USA) and polyoxyethylene-(40)-stearate (1mg/ml; Sigma) that was gas-saturated. For targeting of microbubbles to leukocytes (MB_Lc_), distearoyl-phosphatidylserine (0.3mg/ml; Avanti Polar Lipids) was added to the suspension before sonication [8]. For targeting of P-Selectin (MB_PSel_) and CD4 (MB_CD4_), distearoyl-phosphatidylethanolamine-PEG(3400)-biotin (0.14mg/ml; Creative PEG Works) was added to the suspension. Anti-P-Selectin antibody (RB40.34) or anti-CD4 antibody (H129.19) were then conjugated to the microbubble shell using biotin-streptavidin linking as previously described [9]. Control microbubbles (MB_Iso_) with a non-specific control antibody of the same isotype (R3-34) were also prepared. We have previously shown that these protocols result in microbubbles of similar mean sizes of 2.5 to 2.7μm [7].

### Animal instrumentation

21 days after induction of EAM, the mice were anesthetized with inhaled isoflurane. The animal core temperature was maintained at 37°C. The chest and neck was depilated. For microbubble injections, the right internal jugular vein was cannulated using PE 50 tubing under sterile conditions and the animals were transferred onto a temperature controlled imaging stage (Vevo Imaging Station). After high resolution echocardiography and CEU molecular imaging, the cannula was withdrawn, the right internal jugular vein was ligated and the wound was closed surgically. Subsequently, the mice were recovered. 60–65 days after induction of EAM, the mice were again anesthetized. High resolution echocardiography was performed. Subsequently, the right common carotid artery was dissected free from surrounding tissue. A 0.33 mm diameter manometer tipped catheter (PVR-1035, Millar, Oxford, England) was inserted through a small incision in the artery. The catheter was then carefully advanced through the aortic valve into the left ventricle. Pressure parameters were recorded and analyzed with a MPVS System (AD Instruments, Dunedin, New Zealand) and included peak first derivative of pressure increase in the left ventricle (dp/dt max), peak first derivative of pressure decrease in the left ventricle (dp/dt min), the left ventricular relaxation time constant Tau, and left ventricular end-diastolic pressure (LVEDP). The mice were then sacrificed and the heart was harvested and preserved in 4% paraformaldehyde for histology.

### Echocardiography

Left ventricular structure and function was assessed using high frequency (40MHz, MS 550D transducer) ultrasound imaging (Vevo 2100, VisualSonics Inc., Toronto, Canada). M-Mode images of the left ventricle at the mid-ventricular level in short-axis orientation were used to measure left ventricular chamber dimensions (LVIDd, left ventricular inner diameter in diastole; LVIDs, left ventricular inner diameter in systole; LVAWd, left ventricular anterior wall diameter in diastole; PWd, left ventricular posterior wall in diastole. Left ventricular mass as well as ejection fraction and volumes were measured from long- and short axis B-mode images using the Vevo 2100 software package version 1.6.0 with the cardiac package LV trace tool. Stroke volume was derived by subtracting left ventricular end systolic volume from the left ventricular end diastole volume. Cardiac output (CO) was calculated by multiplying the stroke volume with the heart rate. Fractional shortening (FS) was calculated as ((LVIDd-LVIDs)/LVIDd)*100. E-wave and A-wave velocity were measured using pulsed wave Doppler tracings from a modified apical 4-chamber view to assess flow across the mitral valve. Parasternal long- and short-axis B-mode images were acquired at a frame rate of >200 frames per second. Longitudinal, radial and circumferential global peak strain values were derived from the B-mode images using speckle tracking software (VevoStrain, VisualSonics) [10]. For this purpose, cine-loops with two cardiac cycles without respiratory motion were selected and the endocardial and epicardial borders were traced on end-diastolic frames. After automated tracking, manual adjustments were made as necessary throughout the cardiac cycles. The strain measurements were then averaged over the two cardiac cycles without the use of temporal smoothing filters. All echocardiographic images were acquired and analyzed by the same investigator (L.X.) who was blinded to all other experimental data. For the assessment of interobserver variability, 20 randomly selected datasets were re-analysed by an investigator (B.A.K.) blinded for previous measurements, and for intraobserver variability, 10 randomly selected datasets were re-analyzed by L.X.

### Contrast enhanced ultrasound molecular imaging

CEU molecular imaging (Sequoia Acuson C512; Siemens Medical Systems) was performed with a high-frequency linear-array probe (15L8). The left ventricle was imaged in a short axis orientation at the papillary muscle level. ECG-gated triggering was used for acquiring end-systolic frames. For the detection of the microbubble contrast agent, a combination of power modulation and pulse inversion imaging with a 7 MHz fundamental frequency with dynamic range set to 50 dB was used. The 15L8 probe and the imaging parameters with a relatively low transmit frequency were employed to maximize sensitivity for the microbubble contrast agent. The gain settings were adjusted below visible speckle and were kept throughout CEU imaging. MB_Is_, MB_Lc_, MB_PSel_, and MB_CD4_ (5x10^6^ microbubbles per injection) were injected in random order, 9 animals received all 4 microbubble preparations, while 20 animals received 3, 17 animals 2 and 8 animals 1 microbubble preparation, respectively. Of note, due to constraints in terms of intravenous volume application, it was not possible to inject each microbubble species in every animal. Ultrasound imaging was stopped from injection until 8 minutes later when imaging was restarted at a mechanical index of 0.87. Time after injection was derived empirically from prior studies and was optimized to achieve minimal background signal from circulating microbubbles while retaining a maximum of targeted signal. The first image frame was used to determine the total amount of microbubbles within the left ventricular myocardial tissue. The microbubbles in the ultrasound field were then destroyed with several (>10) image frames. Several image frames at a long pulsing interval (every 9 heartbeats) were subsequently acquired to measured signal from circulating microbubbles. After log-linear conversion, frames depicting circulating microbubbles were subtracted from the initial image to compute signal from attached microbubbles. Contrast intensity was measured from a region of interest containing the whole left ventricular myocardium. The region of interest was selected using fundamental frequency anatomic images of the left ventricle imaged at 14MHz. Contrast enhanced ultrasound imaging data were analyzed blinded to all other experimental data by one investigator (D.C.S.). For the assessment of interobserver variability, a total of 40 randomly selected datasets (10 from each microbubble preparation) and for intraobserver variability, 10 randomly selected datasets were reanalyzed by an investigator blinded to previous data (M.P.).

### Histology

For the quantification of myocardial fibrosis, heart tissue was fixed in 4% formaldehyde-solution and 5 um thin short axis sections of the left ventricle were prepared using a water-slide microtome (HM 340E, Zeiss, Jena). Two 5 μm sections approximately 250 μm apart where the papillary muscles were visible were stained and analyzed per animal. The sections were stained with PicroSirius Red and counter-stained with hematoxylin. Full-spectrum RGB images of the stained myocardium were recorded (NikonTi microscope, Plan Apo λ 10X objective). The Nikon NIS-Elements software was used for image analysis. User defined low, intermediate and high % fibrotic sections were used to establish two thresholds using the hue, intensity and saturation (HIS) features of the images. The first threshold was aimed to include the entire tissue. The second threshold was devised to comprise the red stained regions. These two thresholds were then applied to the entire data set. On each section a region of interest was defined in the left ventricle and measurements were only performed on that region. Fibrosis % is the thresholded red signal normalized to the thresholded entire LV tissue. The average of the two measurements was used for further analysis. Myocardial fibrosis quantification was performed blinded to all other experimental data by one investigator (A.O).

### Statistical analysis

GraphPad Prism (version 6.07) was used for statistical analysis. Data are expressed as median (25th - 75th percentile). Comparisons between data at day 21 and day 63 after induction of EAM were analyzed using a Wilcoxon matched-pair rank test. For correlations between 2 parameters (e.g. fibrosis score vs. functional parameters), Spearman correlation was used. A two-sided p value <0.05 was considered as statistically significant. Inter- and intra-observer variability was evaluated using Bland-Altman analysis.

## Results

### Physiological and echocardiographic parameters on day 21 and day 63

Physiologic and echocardiographic measurements of left ventricular dimensions are shown in Table 1. The bias (95% limits of agreement) values were calculated for interobserver variability for left ventricular volume in diastole, left ventricular volume in systole, left ventricular ejection fraction, cardiac output and fractional shortening as 0.7222 (-5.470 to 6.915), 1.315 (-4.926 to 7.556), -1.249 (-8.414 to 5.916), -0.5635 (-5.341 to 4.214), -3.707 (-10.34 to 2.923) respectively. For intraobserver variability the values were -4.177 (-7.804 to -0.5506), -1.879 (-5.912 to 2.155), -0.1489 (-6.150 to 5.852), -1.007 (-3.429 to 1.416), 0.8216 (-2.938 to 4.581) respectively. The induction of EAM lead to a decrease in heart rate, possibly due to augmented cardiac depression during isoflurane anesthesia with an increase in cavity dimensions and a decrease in myocardial wall thickness, while calculated left ventricular mass remained unchanged. Left ventricular cavity volumes remained increased on day 63 when correcting for the increase in bodyweight (2.1 (1.9–2.5) μl/g vs 2.8 (2.6–3.1) μl/g, p<0.0001). Thus, EAM resulted in eccentric left ventricular remodeling without development of hypertrophy.

**Table 1:** Physiologic data and echocardiographic parameters of left ventricular dimensions.

	Day 21	Day 63	p-value
**Bodyweight (g)**	21.1 (19.2–21.6)	22.7 (21.9–23.6)	<0.0001
**Heart rate (bpm)**	421 (391–452)	364 (345–389)	<0.0001
**Heart weight**	-	133 (127–144)	-
**LV AWd (mm)**	0.91 (0.83–1.06)	0.85 (0.77–0.96)	0.05
**LV IDd (mm)**	3.50 (3.20–3.73)	3.90 (3.84–4.02)	<0.0001
**LV PWd (mm)**	0.75 (0.71–0.90)	0.68 (0.63–0.72)	<0.0001
**LV IDs (mm)**	2.10 (1.78–2.42)	2.87 (2.76–3.16)	<0.0001
**LV mass (mg)**	90 (82.98–99.9)	86 (81.5–94.2)	ns
**LV Vold (μl)**	58.8 (51.9–65.2)	75.6 (67.9–79.7)	<0.0001
**LV Vols (μl)**	20 (15.3–24.2)	39.7 (33.9–45.6)	<0.0001

bpm, beats per minute, LV AW_d_ left ventricular anterior wall in diastole, LV ID_d_ left ventricular inner diameter in diastole, LV PW_d_ left ventricular posterior wall in diastole, LV ID_s_ left ventricular inner diameter in systole, LV mass left ventricular mass, LV Vol_d_ left ventricular volume in diastole, LV Vol_s_ left ventricular volume in systole

Left ventricular systolic function and cardiac output was reduced on day 63 after induction of EAM (Table 2). This was true for global left ventricular ejection fraction as well as for speckle-tracking derived myocardial strain and strain rate which were reduced in the longitudinal, circumferential and radial dimension. Diastolic function was also affected with a highly significant change in E/A ratio due to a decrease in A wave velocity.

**Table 2:** Echocardiographic parameters of systolic and diastolic function.

	Day 21	Day 63	p-value
**Ejection fraction (%)**	67 (59.2–73.9)	47.2 (42–51.6)	<0.0001
**Cardiac output (ml/min)**	16 (14–18)	14 (11–15)	<0.0001
**Fractional shortening (%)**	15 (11–19)	11 (10–14)	<0.0001
**Longitudinal strain (%)**	8.7 (7.4–10.2)	6.9 (5.8–8.0)	<0.001
**Circumferential strain (%)**	-18 (-22 –-16)	-13 (-15 –-10)	<0.0001
**Radial strain (%)**	33 (25–42)	24 (19–27)	<0.0001
**Longitudinal strain rate (s**^**-1**^**)**	-5.6 (-6.8 –-5.1)	-4.4 (-4.7 –-3.7)	<0.001
**Circumferential strain rate (s**^**-1**^**)**	-13 (-15 –-11)	-11 (-12 –-10)	<0.0001
**Radial strain rate (s**^**-1**^**)**	-7.6 (-8.4 –-6.1)	-4.5 (-5.1 –-3.4)	<0.0001
**E wave velocity (cm/s)**	67 (59–74)	65 (56–81)	ns
**A wave velocity (cm/s)**	31 (18–36)	15 (0.5–21)	<0.0001
**E/A ratio**	1.9 (1.6–2.1)	4.0 (2.8–6.0)	<0.0001

### Invasive hemodynamics on day 63

Due to the invasive nature of left ventricular pressure measurements, these parameters were assessed on day 63 only, and thus values are compared to published normal values rather than to values assessed on day 21 (Table 3). However, compared to published normal values an increase in left ventricular end diastolic pressure and a slowing in left ventricular pressure decay during early diastole were evident, indicating diastolic dysfunction in addition to systolic dysfunction evidenced using echocardiography.

**Table 3:** Invasive hemodynamics–comparison to published normal values [11].

	Day 63	Normal
**dp/dt max (mmHg/s)**	6597 (5518–7245)	7700–14480
**dp/dt min (-mmHg/s)**	5987 (6968–5102)	6900–10400
**Tau (ms)**	12.4 (9.6–14.3)	4.4–7.6
**LVEDP (mmHg)**	12.5 (8.2–17.0)	1–6

dp/dt max, peak first derivative of pressure increase in the left ventricle; dp/dt min, peak first derivative of pressure decrease in the left ventricle; Tau, left ventricular relaxation time constant; LVEDP, left ventricular enddiastolic pressure.

### Development of myocardial fibrosis on day 63

**Fig 1:**
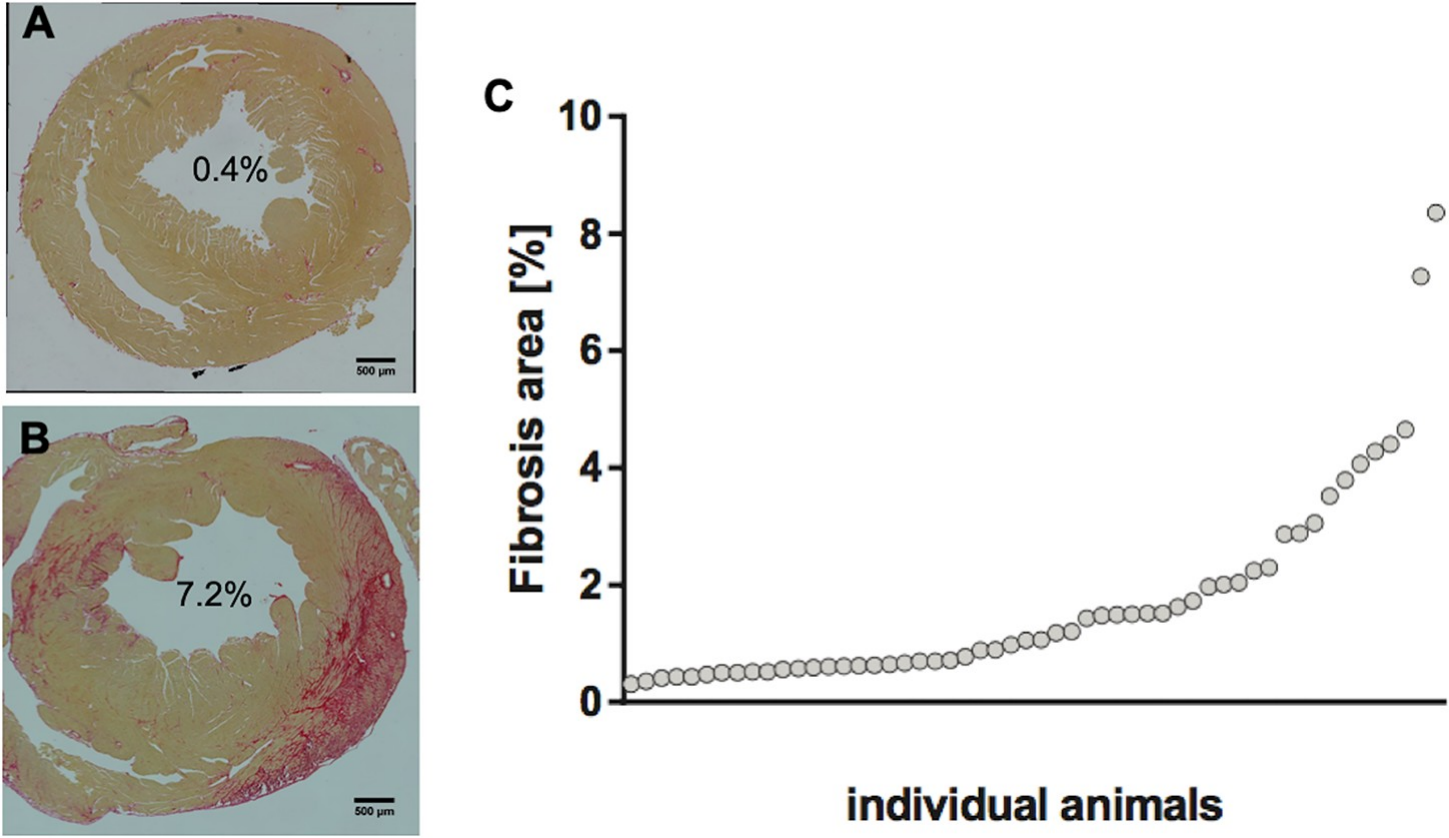
Extent of fibrosis as assessed on PicroSirius Red stained histological sections. (A) demonstrates an example of minimal fibrosis, (B) an example of extensive fibrosis both in the anterolateral and septal left ventricular wall. (C) graph illustrating the distribution of the extent of fibrosis among all animals included in the study.

Quantification of collagen using PicroSirius Red staining showed degrees of fibrosis ranging from 0.3 to 8.4% of the total myocardial area (Fig 1). Myocardial fibrosis was observed both in the left ventricular and right ventricular myocardium with predominant involvement of the subepicardium. 49% of the animals showed <1%, 24% 1–2% and 27% >2% of myocardial fibrosis.

### Correlation of ultrasound molecular imaging with extent of fibrosis and functional data on day 63

**Fig 2:**
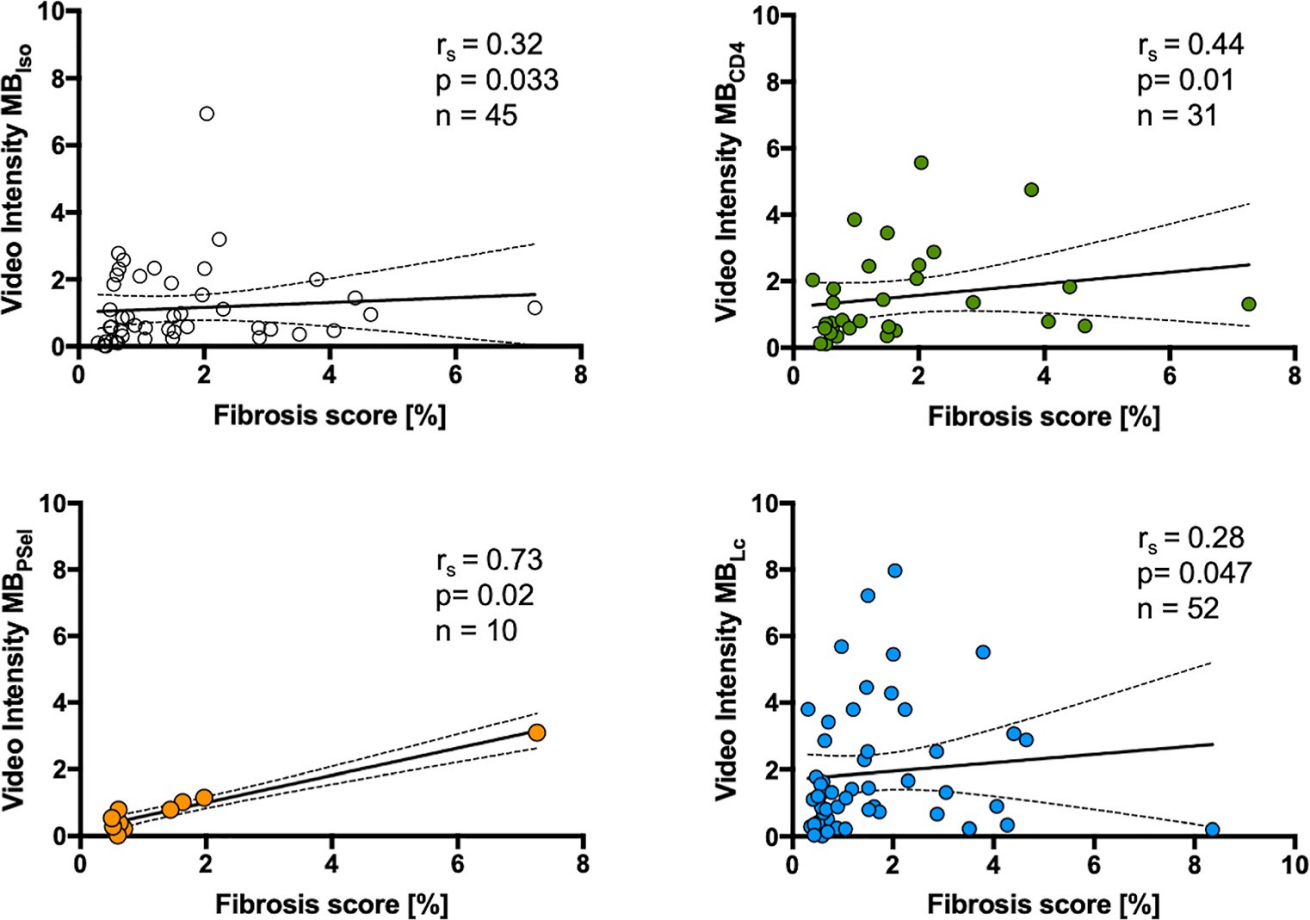
Correlation of ultrasound molecular imaging on day 21 with degree of fibrosis on day 63. r_s_ denotes Spearman r.

The bias (95% limits of agreement) value was calculated for interobserver variability for ultrasound molecular imaging data as -0.02178 (-1.014 to 0.975), while for intraobserver variability the value was -0.06610 (-0.2584 to 0.1262). Correlations of signal from microbubbles targeted to leukocytes, to CD4+ lymphocytes and to P-Selectin as well as for control microbubbles are shown in Fig 2. Signal from all microbubble species did correlate significantly with the extent of fibrosis. However, this correlation was weak for control microbubbles and for microbubbles targeted to leukocytes and intermediate for microbubbles targeted to CD4+ lymphocytes. Correlation was best for microbubbles targeted to P-Selectin, however this correlation was based on fewer data points. Also, there was a weak but significant correlation between MB_Lc_, signal and LVEF on day 63 (r = -0.28, p = 0.048). In contrast, there was no correlation between signals from microbubbles versus all other functional parameters on day 63 (MB_Iso_, MB_Lc_, MB_CD4+_, MB_PSel_ versus longitudinal strain, circumferential strain, radial strain, LVEDP, Tau, dp/dt max, dp/dt min; all nonsignificant).

### Correlation of functional data on day 21 with extent of fibrosis on day 63

Correlations between functional parameters on day 21 and the extent of fibrosis on day 63 are shown in Table 4. There was a significant correlation only for left ventricular mass and E wave velocity with later extent of fibrosis.

**Table 4:** Correlation of functional parameters on day 21 with extent of fibrosis on day 63.

Parameter vs. fibrosis	r	p	n
**Ejection fraction (%)**	-0.27	0.053	53
**Cardiac output (ml/min)**	-0.13	0.346	53
**Fractional shortening (%)**	-0.27	0.051	53
**LV Vold (μl)**	-0.07	0.60	53
**LV Vols (μl)**	0.17	0.24	53
**LV Mass (mg)**	0.48	0.0003	53
**Longitudinal strain (%)**	0.24	0.08	53
**Circumferential strain (%)**	0.22	0.11	54
**Radial strain (%)**	-0.16	0.25	53
**E wave velocity (cm/s)**	0.29	0.04	52
**A wave velocity (cm/s)**	-0.06	0.68	52
**E/A**	0.12	0.40	53

## Discussion

Myocarditis and the ensuing autoimmune processes lead to structural damage with myocardial fibrosis, loss of tissue architecture, and impairment in diastolic and systolic function. Thus, myocarditis can ultimately lead to DCMP with heart failure, risk for sudden death and the need for heart transplantation. The clinical presentation of myocarditis varies widely. Estimates of the rate of progression to DCMP derived from prospective studies are lacking. However, prospective studies show that during follow-up patients with a diagnosis of acute myocarditis experience sudden cardiac death or heart transplant in 4–20%, suggesting a rate of progression to DCMP in at least that range [2,12]. Therefore, a non-invasive imaging method that adds prognostic information regarding the risk of progression to DCMP would be a useful adjunct to diagnostic tools for assessing patients with myocarditis. The results of our study indicate that contrast enhanced ultrasound molecular imaging of inflammatory cell infiltration or endothelial activation does correlate to the development of myocardial fibrosis. Also, there was a weak correlation between leukocyte targeted imaging and ejection fraction on day 63. However, we did not see a correlation between the results of ultrasound molecular imaging and other parameters of left ventricular function. However, the spread of ultrasound molecular imaging signals was considerable when compared to the ultimate development of fibrosis. Therefore, ultrasound molecular imaging of inflammatory cell recruitment and endothelial activation at a single timepoint during acute myocarditis and methodological weaknesses may account for the limited potential of the technique for the prediction of later structural and functional consequences.

Experimental autoimmune myocarditis has extensively been used to study the pathophysiology of autoimmune processes that follow acute viral myocarditis. In our study, cardiac myosin induced autoimmune myocarditis lead to a phenotype of DCMP after 63 days. Thus, on high frequency echocardiography, the left ventricle was dilated, LVEF was decreased, and an increased E/A suggested diastolic dysfunction. This was supported by invasive hemodynamics showing an increase in the left ventricular relaxation time constant Tau indicating impaired relaxation and an increased left ventricular filling pressure, as well as increased myocardial fibrosis. As was to be expected from reported rates of successful induction of EAM in female balb/c mice of around 60% [6], there was a wide range of percentage of myocardial fibrosis and reduction in left ventricular systolic and diastolic function and a subset of animals did not show features of DCMP. Despite this wide range of pathologic changes induced in the animals that were examined with contrast enhanced molecular imaging, assessment of subsets of the inflammatory response with ultrasound molecular imaging were only weakly predictive of development of fibrosis and were not able to predict functional changes.

There are a number of reasons that may explain the low predictive value of ultrasound molecular imaging in this particular setting. First, the autoinflammatory process which is of a low-grade intensity in the majority of mice induced [7] was sampled at a single timepoint, this may have failed to pick up the effects of an ongoing low grade inflammation on development of DCMP. Second, longitudinal 18F-FDG PET imaging in rats has shown that peak inflammation in autoimmune myocarditis occurs around 3 weeks after induction, with a sharp decline in inflammatory activity thereafter, and thus small differences in timing of imaging at a single timepoint might potentially result in large differences in inflammatory activity recorded [13]. Third, while the acute inflammatory response in EAM depends on an adaptive immune response mediated by T-lymphocytes, it has been shown that progression to DCMP is critically dependent on secretion of interleukin 17A by Th17 lymphocytes, and thus targeting of this lymphocyte subset may yield a more precise information regarding subsequent ventricular remodeling [14]. Last, the stochastic nature of ultrasound together with the need to image microbubble retention in the myocardium at high heart rates at a relatively low imaging frequency with limited spatial resolution without the possibility of customizing the pulse inversion algorithm for the microbubble in use and in the presence of respiratory motion may have induced variability in signal that precluded a better prediction of ventricular remodeling. In terms of variability of the ultrasound data, we present interobserver and intraobserver variability data from single datasets, however, acquisition of repeated datasets in the sense of test-retest variability could have shed further light on the reliability of the ultrasound data and would have been optimal. Also, the results that we report apply to the particular EAM animal model used, while the pathogenesis of myocarditis varies and therefore the predictive value of molecular imaging of inflammatory phenotypes may be different.
